# A stratified compartmental model for the transmission of *Sparicotyle chrysophrii* (Platyhelminthes: Monogenea) in gilthead seabream (*Sparus aurata*) fish farms

**DOI:** 10.1098/rsos.221377

**Published:** 2023-05-17

**Authors:** Elisa Stella, Roberto Pastres, Damiano Pasetto, Matko Kolega, Danijel Mejdandžić, Slavica Čolak, Antares Musmanno, Andrea Gustinelli, Lorenzo Mari, Enrico Bertuzzo

**Affiliations:** ^1^ Department of Environmental Sciences, Informatics and Statistics, Ca’ Foscari University of Venice, 30123 Venice, Italy; ^2^ Cromaris D.D., 23000 Zadar, Croatia; ^3^ Department of Veterinary Medical Sciences, Alma Mater Studiorum Università di Bologna, 40064 Bologna, Italy; ^4^ Dipartimento di Elettronica, Informazione e Bioingegneria, Politecnico di Milano, 20133 Milano, Italy

**Keywords:** epidemiological model, gilthead seabream, parasites, epizootics, aquaculture

## Abstract

The rapid development of intensive fish farming has been associated with the spreading of infectious diseases, pathogens and parasites. One such parasite is *Sparicotyle chrysophrii* (Platyhelminthes: Monogenea), which commonly infects cultured gilthead seabream (*Sparus aurata*)—a vital species in Mediterranean aquaculture. The parasite attaches to fish gills and can cause epizootics in sea cages with relevant consequences for fish health and associated economic losses for fish farmers. In this study, a novel stratified compartmental epidemiological model of *S. chrysophrii* transmission was developed and analysed. The model accounts for the temporal progression of the number of juvenile and adult parasites attached to each fish, as well as the abundance of eggs and oncomiracidia. We applied the model to data collected in a seabream farm, where the fish population and the number of adult parasites attached to fish gills were closely monitored in six different cages for 10 months. The model successfully replicated the temporal dynamics of the distribution of the parasite abundance within fish hosts and simulated the effects of environmental factors, such as water temperature, on the transmission dynamics. The findings highlight the potential of modelling tools for farming management, aiding in the prevention and control of *S. chrysophrii* infections in Mediterranean aquaculture.

## 1. Introduction

Aquaculture has experienced steady growth in global fish production in recent decades and is expected to expand further in the coming years, also in view of the world’s growing population [1]. In Europe, aquaculture contributed to 10% of the total fish production in 2020 [1]. However, Mediterranean aquaculture is also known to bear negative impacts on the environment and the marine habitat [2], impairing water and bottom sediment quality [3–5]. In addition, the expansion of fish farming in cages has been linked to the spread of infectious diseases, pathogens and parasites [6].

Gilthead seabream is the most produced species in European marine finfish aquaculture (36% of total production, 88 000 tons yr^−1^), with an estimated economic value of €473 million [7]. However, one of the challenges facing seabream farmers in the region is the emergence of infections of *Sparicotyle chrysophrii*, a flatworm parasite of the phylum Platyhelminthes. This ectoparasitic monogean flatworm attaches to fish gills and can cause lethal epizootics in sea cages [8] due to its blood-feeding activity. Severe infection can lead to hypoxia and anaemia, which in turn may induce a state of lethargy. The parasite also causes haemorrhages and necrosis [9–11]. Moreover, infected fish are more susceptible to secondary bacterial, viral or parasitic infections due to their compromised immune system [12]. The outbreak of *S. chrysophrii* can thus have important economic consequences for seabream farming.

*S. chrysophrii* is a hermaphrodite parasite. Once it releases eggs, these can either directly remain attached to the host or be released into the environment. In an intensive fish-farm setting, free-floating eggs can attach to cage nets, thus spatially constraining the transmission cycle and likely increasing infection rates. After hatching, the oncomiracidia (free-swimming ciliated larvae) can survive in the water column up to a couple of days [13] while seeking for a fish host. Previous studies showed how infections depend also on environmental factors, in particular on water temperature [8,14]. However, the literature reports contrasting effects of this variable on the parasite life cycle: warm temperature promotes fast parasite development, accelerating the spreading of infections [14], although disease outbreaks have been reported also in winter, when gilthead seabream are more immunodepressed, and, accordingly, more susceptible to the infection [8]. Furthermore, it has been reported that the parasite life cycle could be influenced by fish size and age [8].

Fish farmers routinely perform parasite surveillance and treatment when necessary. Surveillance is typically conducted through gill inspection focused on *S. chrysophrii* detection, while treatment commonly relies on anti-parasitic baths (usually with formalin, where permitted, or other suitable compounds) [15,16]. Other intervention strategies to limit parasite transmission are based on the substitution or cleaning of cage nets to avoid the trapping of parasite eggs. Alternative strategies against *S. chrysophrii* consist in functional feeding [17–19] aimed at preventing and/or treating against infection through dietary additives. Regardless of the type of action taken to treat the fish, to be effective, control efforts have to take into account the parasite life cycle, environmental conditions and infection dynamics [13,14,20].

In this context, epidemiological modelling potentially represents a valid tool to help fish farmers understand parasite transmission and, accordingly, design control measures. To our knowledge, no modelling studies exist on *S. chrysophrii* transmission. For this purpose, in the following, we propose and analyse a novel epidemiological model that investigates *S. chrysophrii* transmission within gilthead seabream farms. We propose a stratified compartmental model, taking into account parasite life cycle, environmental variables and aquaculture practices. This kind of approach has already been adopted in other studies on parasite infection dynamics [21–24] and can be adapted depending on the object of study and the epidemiological dynamics involved. We then applied the model to data collected in a seabream farm managed by Cromaris (Bisage, Croatia).

## 2. Stratified compartmental model for *S. chrysophrii*

We developed the so-called stratified compartmental model [21–24] to reproduce the life cycle of the parasite and the distribution of the abundance of parasite in each fish host. To account for the major pathogenicity and fertility of adult parasites, we differentiate the parasites in two classes: juveniles (attached larvae) and adults. As a result, the fish population is actually subject to a double stratification based on the abundance of juvenile and adult parasites hosted by each individual. Specifically, we define *X*_*j*,*a*_ as the number of fish with respectively *j* juvenile and *a* adult parasites attached, with *j* = 0, 1, 2, …, *J* and *a* = 0, 1, 2, …, *A* (where *J* and *A* are the maximum number of juvenile and adult parasites, respectively). Approximating *X*_*j*,*a*_ as a continuous function of time, *X*_*j*,*a*_(*t*), its dynamics for 0 < *j* < *J* and 0 < *a* < *A* can be written as follows:

2.1 $$\begin{array}{rl} {\dot{X}}_{\;j,a} & =FX_{\;j-1,a}+(j+1)rX_{\;j+1,a-1}+(j+1)\mu_{j}X_{\;j+1,a}+(a+1)\mu_{a}X_{\;j,a+1}\\ & \;+-(F+jr+j\mu_{j}+a\mu_{a}+\mu_{F}+a\mu_{F^{\prime }})X_{\;j,a}. \end{array}$$

**Figure 1.**
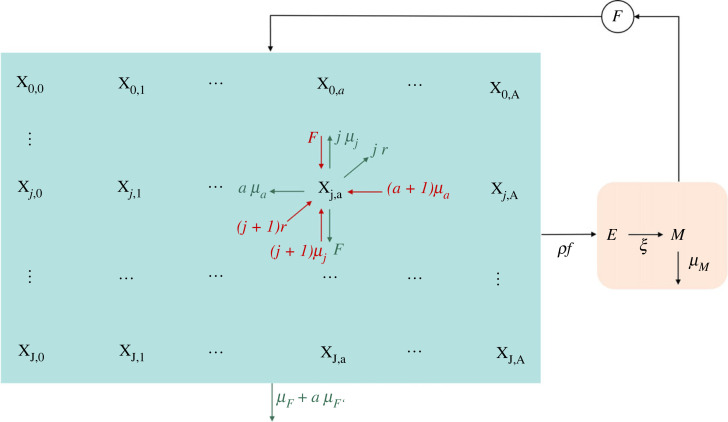
Conceptual diagram of the *S. chrysophrii* transmission model. In the fish compartments, **X** (light blue box), we distinguish between input (in dark red) and output (in green) flows. Parasite compartments, *E* and *M*, are shown in the light pink box.

Equation (2.1) accounts for four input and six output fluxes, which are represented in figure 1 and detailed in the following. A parasite larva that attaches to a fish host, with *j* − 1 juvenile larvae attached, causes an instantaneous increase of *X*_*j*,*a*_ and a related decrease of *X*_*j*−1,*a*_. The rate of such process is *F* = *βM*/*V*, where *β* is the exposure rate and *M* is the number of oncomiracidia within a control volume *V*. A juvenile parasite becomes adult at a rate *r*. Thus, in a fish hosting *j* + 1 juveniles, the rate at which one among the *j* + 1 juveniles become adult is *r*(*j* + 1), which is thus the rate of the transition from *X*_*j*+1,*a*−1_ to *X*_*j*,*a*_. Juvenile parasites die at rate *μ*_*j*_, whilst adults die at a rate *μ*_*a*_. Similarly to the rationale introduced for parasite maturation, the rate at which one of the *j* juvenile (or *a* adult) parasite dies is *μ*_*j*_*j* (or *μ*_*a*_*a*). The latter rates accounts for the transitions *X*_*j*+1,*a*_ → *X*_*j*,*a*_ and *X*_*j*,*a*+1_ → *X*_*j*,*a*_ reported as input terms in equation (2.1). Output fluxes from the state variable *X*_*j*,*a*_ are due to fish mortality, at a rate *μ*_*F*_, and fish excess mortality due to the parasite. The latter is assumed to be linearly proportional to the adult parasite abundance, *aμ*_*F*′_, while extra-mortality possibly linked to the presence of juvenile stages is considered to be negligible. The remaining four negative fluxes reported in equation (2.1) (with overall rate: *F* + *jr* + *jμ*_*j*_ + *aμ*_*a*_) represent the output fluxes corresponding to the four input fluxes described earlier. The fluxes involving state transitions are described in table 1.

**Table 1.** Compartment transitions and rates involving as a target the element *X*_*j*,*a*_ of the fish matrix, **X**. Notice that fish death rates, *μ*_*F*_ and *μ*_*F*′_, are not included, since they do not imply a transition between compartments, causing rather a net loss.

event	event rate	transition
parasite transmission	*F*	*X*_*j*−1,*a*_ → *X*_*j*,*a*_
death of an adult parasite	(*a* + 1)*μ*_*a*_	*X*_*j*,*a*+1_ → *X*_*j*,*a*_
death of a juvenile parasite	(*j* + 1)*μ*_*j*_	*X*_*j*+1,*a*_ → *X*_*j*,*a*_
juvenile parasite maturation	(*j* + 1)*r*	*X*_*j*+1,*a*−1_ → *X*_*j*,*a*_

If we imagine that the state variables *X*_*j*,*a*_ are arranged in a matrix, where rows and columns represent juvenile and adult parasite abundance, respectively, special care has to be devoted to the equations representing the dynamics of the state variables at the corners of the matrix, i.e. having the maximum or minimum number of juveniles and adults (*j* = 0, *J* and *a* = 0, *A*) (equations (2.2)–(2.5)):

2.2 $$\;{\dot{X}}_{0,0}=\mu_{j}X_{1,0}+\mu_{a}X_{0,1}-(\mu_{F}+F)X_{0,0,}$$

2.3 $$\;{\dot{X}}_{J,0}=FX_{J-1,0}+\mu_{a}X_{J,1}-(Jr+J\mu_{j}+\mu_{F})X_{J,0,}$$

2.4 $$\;{\dot{X}}_{0,A}=rX_{1,A-1}+\mu_{j}X_{1,A}-(F+A\mu_{a}+\mu_{F}+A\mu_{F^{\prime }})X_{0,A}$$

2.5 $$\mathrm{and}\;\;{\dot{X}}_{J,A}=FX_{J-1,A}-(J\mu_{j}+A\mu_{a}+\mu_{F}+A\mu_{F^{\prime }})X_{J,A}$$

and at the edges (equations (2.6)–(2.9)):

2.6 $${\dot{X}}_{0,a}=rX_{1,a-1}+\mu_{j}X_{1,a}+\mu_{a}X_{0,1}-(F+a\mu_{a}+\mu_{F}+a\mu_{F^{\prime }})X_{0,a},$$

2.7 $${\dot{X}}_{J,a}=FX_{J-1,a}+(a+1)\mu_{a}X_{J,a+1}-(Jr+J\mu_{j}+a\mu_{a}+\mu_{F}+a\mu_{F^{\prime }})X_{J,a},$$

2.8 $${\dot{X}}_{\;j,0}=FX_{\;j-1,0}+(j+1)\mu_{j}x_{\;j+1,0}+\mu_{a}X_{\;j,1}-(F+jr+j\mu_{j}+\mu_{F})X_{\;j,0},$$

2.9 $$\begin{array}{rl} {\dot{X}}_{\;j,A}=FX_{\;j-1,A} & +(j+1)rX_{\;j+1,A-1}+(j+1)\mu_{j}x_{\;j+1,A}\;+-(F+j\mu_{j}+A\mu_{a}+\mu_{F}+A\alpha_{a})X_{\;j,A}, \end{array}$$

which represent the boundary conditions for equation (2.1).

The description of the parasite life cycle is completed by modelling two additional state variables: the number of parasite eggs in the system, *E*, and the number of oncomiracidia, *M*. The equations regulating their dynamics are as follows:

2.10 $$\dot{E}=\rho f\sum_{\;j=0}^{J}\sum_{a=1}^{A}aX_{\;j,a}-\xi E$$

and

2.11 $$\dot{M}=\xi E-F\sum_{\;j=0}^{J-1}\sum_{a=0}^{A}X_{\;j,a}-\mu_{M}M.$$

Each adult parasite can release eggs with a shedding rate *f*, which can survive and hatch with probability *ρ*, for a total amount of $\rho f\sum_{\;j=0}^{J}\sum_{a=1}^{A}aX_{\;j,a}$ eggs released per unit time by the fish population. Eggs hatch into oncomiracidia at rate *ξ*, and eventually find a host, thus being transferred to the fish compartment (second term of the right-hand side of equation (2.11)), or are removed from the cage. The latter process occurs at a rate *μ*_*M*_, which accounts for both oncomiracidia mortality and possible exit from the cage.

In the absence of evidence of density-dependent effects, we assume that parasite growth, infection and death rates are independent of the parasite abundance (i.e. *r*, *μ*_*a*_, *μ*_*j*_, *μ*_*M*_, *f* and *ξ* do not depend on *a* and *j*).

## 3. Case study

### 3.1. Data

We applied the model to data collected in six cages (in the following referred to as cages 1–6) from a seabream farm managed by Cromaris d.d. The cages are located in Bisage, Croatia (44°01′30″ N, 15°13′10″ E), and have a unit volume of about 225 m^3^ each. The experiment started in February 2021 with about 10^4^ fish per cage with an average weight of 8.5 g. A repeated parasitological survey was run between February and November 2021: each month, 30 fish were collected from each cage (1800 total fish sampled) and, for each fish, all eight arch gills were examined to count the number of attached adult parasites. The fish stock size was re-evaluated each month during the experiment. At the end, the number of fish in the cages was approximately 9000, with an average weight of about 287 g. At each sampling, water temperature, *T* (°C), was also measured.

### 3.2. Model setup and calibration

To run the model, we set the maximum number of adult and juvenile parasites, respectively, as *A* = 30 and *J* = 15 individuals. These values do not affect the model output as long as the number of fish that reach the maximum intensity of infection remains close to zero during the simulation. We checked *a posteriori* that such condition was verified in our simulations. The initial population was set to *H* = 10^4^ fish like in the experimental cages. We assumed that the fish mortality parameters, *μ*_*F*_ and *μ*_*F*′_, the exposure and juvenile maturation rates, *β* and *r*, and the hatching rate, *ξ*, possibly vary with temperature following a widely used power-law formulation. Accordingly, we set their reference values at $20^{\circ }C$ (i.e. *μ*_*F*20_, *μ*_*F*′20_, *β*_20_, *r*_20_ and *ξ*_20_, respectively) so that

$$\begin{array}{rl} \mu_{F} & =\mu_{F20}\theta_{\mu_{F}}^{T-20},\\ \mu_{F^{\prime }} & =\mu_{F^{\prime }20}\theta_{\mu_{F}}^{T-20},\\ \beta & =\beta_{20}\theta_{\beta }^{T-20},\\ \xi & =\xi_{20}\theta_{\xi }^{T-20}\\ \mathrm{and}\;\;r & =r_{20}\theta_{r}^{T-20}, \end{array}$$

where the *θ* parameters express the sensitivity of each rate to water temperature.

As initial conditions, we assume that *M*_0_ oncomiracidia are present in the cage while all other state variables are null, except for the uninfected fish abundance. We thus set, as of February 2021 (*t* = 0): *M*(0) = *M*_0_, *E*(0) = 0, *X*_0,0_(0) = *H* and $X_{\;j,a}(0)=0\;\forall j,a>0$.

Parameter estimation relies on a Bayesian framework and is based on Markov chain Monte Carlo (MCMC) sampling. We used the DREAM algorithm (Differential Evolution Adaptive Metropolis) [25], which is an implementation of MCMC that runs multiple chains simultaneously, in order to efficiently explore the parameter space. In particular, we adopted the DREAM_*ZS*_ version [26]. We assign a range of values where the algorithm is allowed to explore parameters up to convergence (a total of $O(3\times 10^{5})$ iterations were run). We also assigned prior Gaussian marginal distributions to those parameters for which literature information is available (table 2).

**Table 2.** Model parameters along with prior and posterior statistics. Prior marginal distributions, where assigned based on literature values, follow a Gaussian distribution with the reported mean and standard deviation.

parameter	units	prior (mean, std)	value or range	references	cage dependent
*μ*_*F*20_	days^−1^	—	10^−5^ to 10^−2^	—	Y
*μ*_*F*′20_	days^−1^	—	0–1/100	—	N
$\theta_{\mu_{F}}$	—	—	0.5–1.5	—	N
*μ*_*a*_	days^−1^	0.1, 0.01	1/100–1/3	[20]	N
*μ*_*j*_	days^−1^	0.1, 0.01	1/100–1/3	—	N
*β*_20_	days^−1^	—	0–2	—	Y
*θ*_*β*_	—	—	0.5–1.5	—	N
*r*_20_	days^−1^	0.03, 0.003	1/100–1/10	[13]	N
*θ*_*r*_	—	—	0.5–1.5	—	N
*ρ* · *f*	days^−1^	—	0–20	—	N
*ξ*_20_	days^−1^	0.15, 0.015	1/20–1/3	[13]	N
*θ*_*ξ*_	—	—	0.5–1.5	—	N
*μ*_*M*_	days^−1^	2, 0.2	0–5	[13]	N
*M*_0_	—	—	0–1.5 × 10^4^	—	Y

To compute the likelihood of the observation we proceeded as follows. From the model simulation, we estimated the time-dependent probability that a randomly sampled fish hosts *a* adult parasites as follows:

3.1 $$p_{a}(t)=\frac{\sum_{\;j=0}^{J}X_{\;j,a}(t)}{\sum_{a=0}^{A}\sum_{\;j=0}^{J}X_{\;j,a}(t)}.$$

Terming *n*_*a*_(*m*_*i*_) the number of fish with *a* adult parasites (a=0,…,A) in the monthly sample *m*_*i*_ of *N* = 30 fish, the log likelihood of a sample can be calculated using a multinomial probability distribution:

3.2 $$\log L(m_{i})=\log N!-\sum_{a=0}^{A}\log n_{a}(m_{i})!+\sum_{a=0}^{A}n_{a}(m_{i})\log p_{a}(t).$$

Finally, the log likelihood of the samples taken at one cage through the growing season is expressed as follows:

3.3 $$\log L=\sum_{m_{i}=1}^{10}L(m_{i}).$$

To better identify the parameters related to fish mortality, we included also data about the number of surviving fish in the likelihood computations. In particular, we assumed that the abundance of fish in a certain month follows a Gaussian distribution with an average equal to the abundance predicted by the model and a standard deviation equal to the one observed among the six cages. The six cages have slightly different epidemic trajectories. These differences could be attributed to slightly different environmental conditions such as net fouling and the related dissolved oxygen concentration. Moreover, different fish biomass growth trajectories, as observed in the data, could affect parasite susceptibility and fish mortality. We therefore allow some specific parameters to possibly assume different values across the different cages. Specifically, they are the mortality rate *μ*_*F*,20_, the exposure rate *β*_20_, and the initial concentration of oncomiracidia *M*_0_. The remaining parameters are instead assumed to the the same for all cages.

## 4. Results

**Figure 2.**
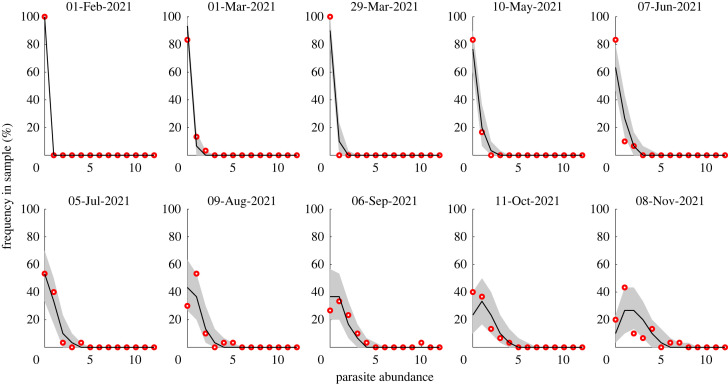
Distribution of adult parasite abundance within fish hosts for the ten monthly samples (red circles indicate data). Here, cage 3 is shown as an example, while the corresponding figures for the other cages are reported in appendix A. Black solid lines and grey shaded areas show the median and the 95% percentile range of the multinomial distribution predicted by the model.

**Figure 3.**
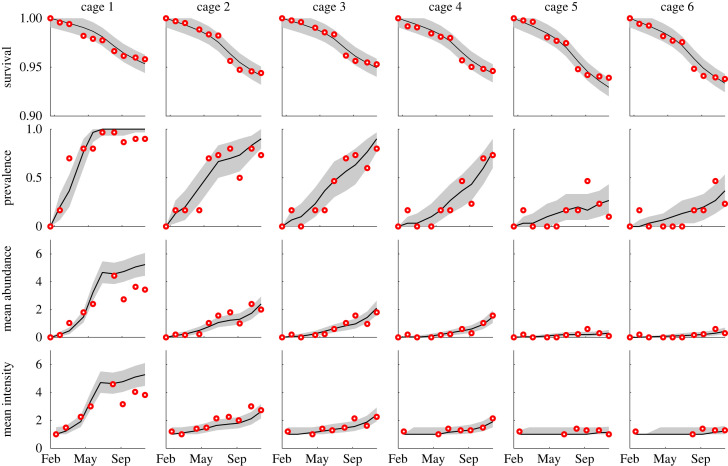
Summary statistics for fish and parasite populations (rows) in the six cages (columns): fraction of fish surviving; prevalence (fraction of fish with at least one parasite); mean parasite abundance (mean number of parasites in the sampled fish); mean parasite intensity (mean number of parasites in infected fish). Red circles show sample data. Black solid lines and grey shaded areas show the median and the 95% percentile range of the model results.

An example of observed distribution of parasite abundance among the sampled fish in a single cage is shown in figure 2. Analogous figures for the remaining five cages are shown in appendix A. At the beginning of the sampling period, all fish have zero parasites. Then, parasite abundance increases, and in summer, the mode of the parasite abundance distribution in the sampled fish reaches one. Figure 3 provides an overview of fish and parasite population statistics. At the end of the sampling period, about 95% of fish survive in all cages, with an observed increase in mortality between July and August. The prevalence, i.e. the fraction of fish with at least one parasite, varies markedly among cages. It is highest in cage 1, in which the share of infected fish reaches more than 60% in April, while the other cages reach high values more gradually, in late spring or early summer. In cages 5 and 6, infected fish prevalence does not exceed 50%. The mean parasite abundance (i.e. the mean number of parasites in the sampled fish [27]) shows a pattern similar to the prevalence, with mean abundance at the end of the period decreasing going from cage 1 to 6. Figure 3 reports the mean intensity, defined as the mean number of parasites in sampled infected fish [27], where the latter are fish hosts with at least one parasite. In cages 5 and 6, infected fish typically have a single parasite, while the mean intensity reached almost five in cage 1. The developed model was able to reproduce the distribution of parasite abundance (figures 2 and 5–9) as well as the main patterns of the summary statistics presented in figure 3.

**Figure 4.**
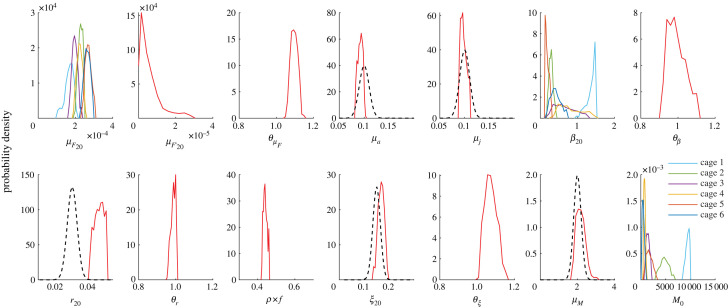
Posterior marginal distributions (coloured lines) and prior marginal distribution (where assigned, black dashed line) of model parameters. For the parameters allowed to vary across cages, the marginal posterior distribution of each cage is shown with different colours (legend).

**Figure 5.**
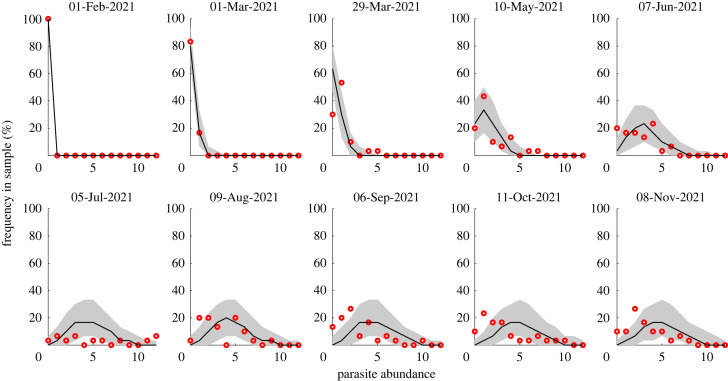
Parasite distribution within fish hosts in cage 1.

**Figure 6.**
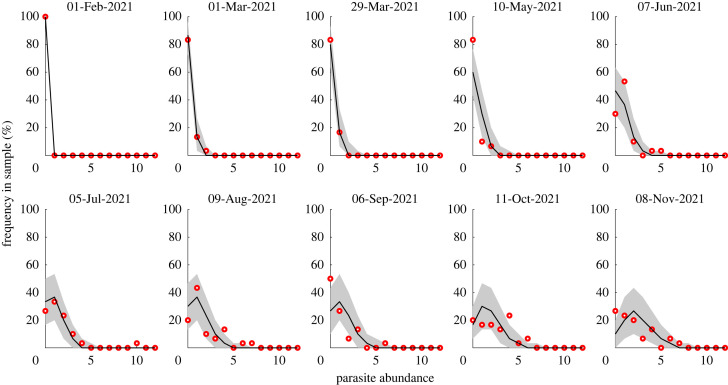
Parasite distribution within fish hosts in cage 2.

**Figure 7.**
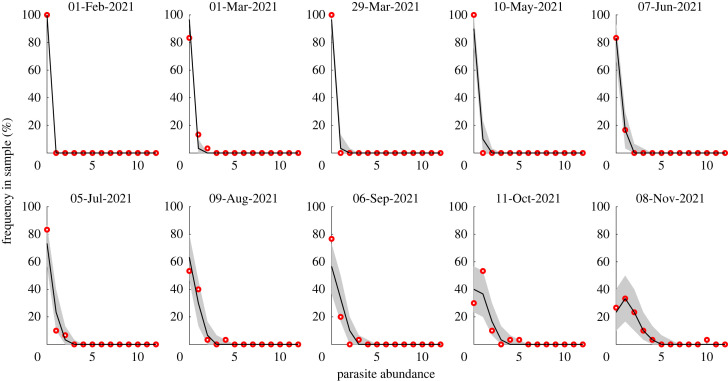
Parasite distribution within fish hosts in cage 4.

**Figure 8.**
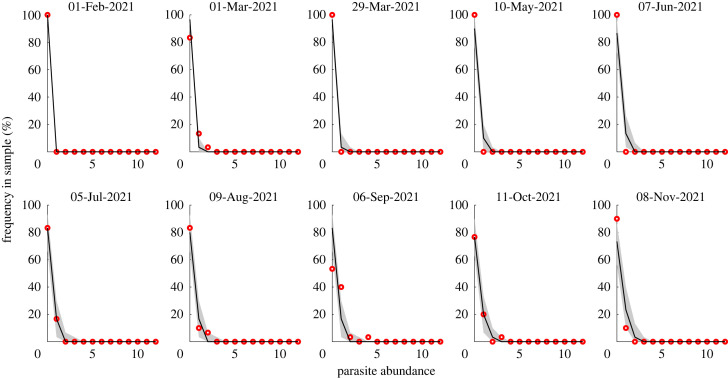
Parasite distribution within fish hosts in cage 5.

**Figure 9.**
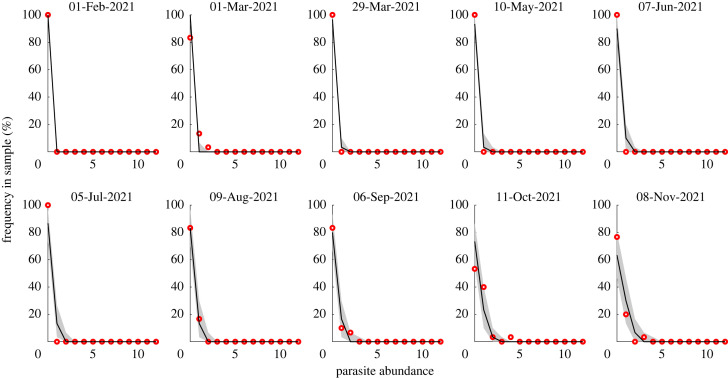
Parasite distribution within fish hosts in cage 6.

The marginal posterior distributions of the estimated parameters (figure 4) enable us to gain further insights into the processes controlling parasite dynamics and to highlight some model limitations. For some parameters, namely, the pathogen juvenile and adult mortality *μ*_*j*_ and *μ*_*a*_, the egg hatching rate *ξ*_20_, and the oncomiracidia loss rate *μ*_*M*_, the posterior distribution shows a good overlap with the prior. This result implies that the dataset does not contain sufficient information to further characterize these process rates, and, in turn, that without prior information, these parameters could not be properly identified. Baseline fish mortality *μ*_*F*_ shows some variability across cages, which leads to the slightly different observed survival trajectories (figure 3), but with a consistent overlap. Results also show that the differences in parasite abundance distribution among the different cages are explained by differences in the exposure rate *β*_20_ and initial conditions *M*_0_. We also note that the posterior sample shows a negative correlation between the exposure rate *β*_20_ and the initial condition *M*_0_ (average correlation coefficient among cages equal to 0.51). This result implies that it is difficult to estimate both parameters, which control the concentration of oncomiracidia in the water, based solely on observations of parasite abundance in fish. Correlation coefficients for the other pairs of parameters are all less than 0.3.

Regarding the effect of temperature on crucial parasite and fish process rates, results indicate that higher temperature enhances fish mortality (median $\theta_{\mu_{F}}=1.10$) and egg hatching rate (median *θ*_*ξ*_ = 1.06). By contrast, for the exposure and parasite maturation rates, the posterior distribution does not allow one to identify a clear temperature effect (i.e. temperature sensitivity parameter close to unity: median *θ*_*β*_ = 0.98, median *θ*_*r*_ = 0.99). As an example, to properly interpret temperature sensitivity values, *θ* = 1.1 implies an increase of 10% of the relevant parameters for every increase of 1°C of water temperature.

Finally, to understand the modelled contribution of parasite infection to overall fish mortality, we run model simulations using the estimated parameters but setting *μ*_*F*′20_ to zero (no parasite-related mortality). Results indicate that the percentage of deaths related to the parasite may range from 6% in cage 1 to 0.1% in cage 6.

## 5. Discussion

In this work, we have proposed a novel mathematical formulation to study the transmission dynamics of *S. chrysophrii* in *S. aurata* aquaculture farms, accounting for the whole life cycle of the parasite. The novelty of the proposed model relies on the double stratification of parasite abundance within the fish host population based on the number of juvenile and adult parasites attached to the gills of each fish. By using this model, we were able to account for the higher pathogenicity of adult parasites, as well as for the link between fish mortality and (adult) parasite abundance. We also included in the model the role of water temperature, which has been found to affect the parasite transmission [8,14]. We applied the model to data obtained from a controlled parasitological survey conducted in *S. aurata* farms located in Bisage (Croatia) and managed by Cromaris d.d. We estimated model parameters using a Bayesian approach that relies on MCMC. Results show that our model reproduces well the available data and the temporal patterns observed in infection dynamics. The results also emphasize the importance of combining modelling studies with laboratory and field experiments to enhance our understanding. In fact, parameter estimation did not provide additional information on some process rates that regulate the parasite’s life cycle, as the posterior distribution overlapped with the prior distribution. Therefore, for the application of the model, it is important to rely on information obtained independently through experimental studies. This will help to improve the accuracy and reliability of the model, particularly in cases where parameter estimation cannot provide sufficient information.

We tested the possible temperature effects on critical parameters that regulate the transmission cycle of *S. chrysophrii*. Our estimation procedure revealed the egg hatching rate *ξ* as temperature dependent, which is consistent with experimental findings [14]. There was no clear evidence to support the temperature dependence of the parasite maturation rate *r* and the exposure rate *β*. However, it should be noted that this could be attributed to limitations in the specific modelling and estimation setup, and further experimental and modelling studies may provide clearer evidence.

Compartmental epidemiological models, like the one formulated in this study, have long been shown to be flexible, yet parsimonious, tools to model a wide array of parasite dynamics and life cycles [21,28,29]. Macroparasitic infections pose specific challenges because the number of parasites within each host matters and should thus be properly accounted for in the model formulation. To this end, stratified compartmental models have been proposed [21–23]. So far, this class of models was developed and tested on human parasitic diseases like schistosomiasis [23,24]. The main challenge in these former applications was that the number of parasites per host could not be directly observed, and therefore the full potential of these tools could not be properly tested. In this regard, the application presented herein offers the ideal setting to test this class of models. Indeed, in farms, the fish population is confined and monitored, and the monthly samples of 30 fish per cage, whose eight arc gills were all examined to count the number of adult ectoparasites, allowed us to reconstruct the distribution of parasite abundance within hosts, which is directly related to the state variables of stratified compartmental models.

One crucial benefit of compartmental models, versus, say, purely statistical models, is that the mechanistic representations of the key processes controlling the parasite life cycle allow one to test the effect of alternative intervention strategies to control the transmission. For instance, the proposed model could be readily adapted to represent treatments like anti-parasitic bath that periodically (or triggered by the surveyed parasite abundance) abate the parasite population in the host. Replacement or cleaning of the cage nets could be represented in the model as a sudden decrease of egg abundance. Net clogging could be represented as a reduction of the flux of eggs that leave the cage. Different intervention strategies, or combinations thereof, could be tested, possibly in an optimal control mathematical framework [30,31], to select the most efficient, accounting also for the ensuing costs.

Infection with *S. chrysophrii* has been reported to reduce the appetite of seabream and affect its metabolism, which in turn affects its growth trajectory. This variable is crucial in fish farming, and a promising avenue for future development is to couple the proposed epidemiological model with a metabolic growth model (e.g. [32–34]). Average fish size or size distribution is commonly monitored in fish farms, and when combined with a well-designed *S. chrysophrii* surveillance system like the one presented here, these data can be used to estimate the parameters of a coupled model that also includes growth dynamics. Such a tool could potentially account for the differential susceptibility of fish of different sizes and the effect of fish biomass density on *S. chrysophrii* transmission, features that cannot be addressed in the current model formulation. We believe that such a tool could more comprehensively account for the full spectrum of the impacts of this parasitic infection and provide a valuable tool for precision fish farming [34].

## Data Availability

Data and code are available through a public repository: https://zenodo.org/record/7236390 [35].

## Appendix A

See figures 5–9.

## References

[1] FAO. 2022 The state of world fisheries and aquaculture (SOFIA). Towards blue transformation. Rome, Italy: FAO.

[2] Casadevall M, Rodríguez-Prieto C, Torres J, Eira C, Marengo M, Lejeune P, Merciai R, Richir J. 2021 Editorial: marine aquaculture impacts on marine biota. Front. Mar. Sci. **8**, 162. (10.3389/fmars.2021.615267)

[3] Morata T, Falco S, Gadea I, Sospedra J, Rodilla M. 2015 Environmental effects of a marine fish farm of gilthead seabream (*Sparus aurata*) in the NW Mediterranean Sea on water column and sediment. Aquac. Res. **46**, 59-74. (10.1111/are.12159) 32313429 PMC7159775

[4] Tsikopoulou I, Moraitis ML, Tsapakis M, Karakassis I. 2018 Can intensive fish farming for 20 years induce changes in benthic ecosystems on a scale of waterbody? An assessment from Cephalonia bay (Ionian Sea). Environ. Monit. Assess. **190**, 469. (10.1007/s10661-018-6846-5) 30019323

[5] Kallitsis E, Korre A, Mousamas D, Avramidis P. 2020 Environmental life cycle assessment of Mediterranean sea bass and sea bream. Sustainability **12**, 9617. (10.3390/su12229617)

[6] Sitjà-Bobadilla A, José Redondo M, Alvarez-Pellitero P. 2010 Occurrence of *Sparicotyle chrysophrii* (Monogenea: Polyopisthocotylea) in gilthead sea bream (*Sparus aurata* L.) from different mariculture systems in Spain. Aquac. Res. **41**, 939-944. (10.1111/j.1365-2109.2009.02369.x)

[7] Guillen J, Virtanen J, Nielsen R, European Commission, Joint Research Centre, Technical Scientific, Economic Committee for 276 Fisheries. 2023 *Economic report on the EU aquaculture (STECF-22-17)*. Luxembourg: Publications Office of the European Union.

[8] Antonelli L, Quilichini Y, Marchand B. 2010 *Sparicotyle chrysophrii* (Van Beneden and Hesse 1863) (Monogenea: polyopisthocotylea) parasite of cultured Gilthead sea bream *Sparus aurata* (Linnaeus 1758) (Pisces: teleostei) from Corsica: ecological and morphological study. Parasitol. Res. **107**, 389-398. (10.1007/s00436-010-1876-0) 20422218

[9] Paperna I, Colorni A, Gordin H, Kissil GW. 1977 Diseases of *Sparus aurata* in marine culture at Elat. Aquaculture **10**, 195-213. (10.1016/0044-8486(77)90001-1)

[10] Sitja-Bobadilla A, Alvarez-Pellitero P. 2009 Experimental transmission of *Sparicotyle chrysophrii* (Monogenea: polyopisthocotylea) to gilthead seabream (*Sparus aurata*) and histopathology of the infection. Folia Parasitol. **56**, 143-151. 10.14411/fp.2009.018 19606789

[11] Henry MA, Nikoloudaki C, Tsigenopoulos C, Rigos G. 2015 Strong effect of long-term *Sparicotyle chrysophrii* infection on the cellular and innate immune responses of gilthead sea bream, *Sparus aurata*. Dev. Comp. Immunol. **51**, 185-193. (10.1016/j.dci.2015.03.010) 25825219

[12] Sitjà-Bobadilla A, Pujalte MJ, Macián MC, Pascual J, Alvarez-Pellitero P, Garay E. 2006 Interactions between bacteria and *Cryptosporidium molnari* in gilthead sea bream (*Sparus aurata*) under farm and laboratory conditions. Vet. Parasitol. **142**, 248-259. 16934406 10.1016/j.vetpar.2006.07.002

[13] Repullés-Albelda A, Holzer AS, Raga JA, Montero FE. 2012 Oncomiracidial development, survival and swimming behaviour of the monogenean *Sparicotyle chrysophrii* (Van Beneden and Hesse, 1863). Aquaculture **338**, 47-55. (10.1016/j.aquaculture.2012.02.003)

[14] Villar-Torres M, Montero FE, Raga JA, Repullés-Albelda A. 2018 Come rain or come shine: environmental effects on the infective stages of *Sparicotyle chrysophrii*, a key pathogen in Mediterranean aquaculture. Parasit. Vectors **11**, 558. (10.1186/s13071-018-3139-3) 30359292 PMC6202810

[15] Leal JF, Neves MGPMS, Santos EBH, Esteves VI. 2018 Use of formalin in intensive aquaculture: properties, application and effects on fish and water quality. Rev. Aquac. **10**, 281-295. (10.1111/raq.12160)

[16] Mladineo I, Trumbić ž, Ormad-García A, Palenzuela O, Sitjà-Bobadilla A, Manuguerra S, Ruiz CE, Messina CM. 2021 In vitro testing of alternative synthetic and natural antiparasitic compounds against the monogenean *Sparicotyle chrysophrii*. Pathogens **10**, 980. (10.3390/pathogens10080980) 34451443 PMC8401465

[17] Rigos G, Fountoulaki E, Cotou E, Dotsika E, Dourala N, Karacostas I. 2013 Tissue distribution and field evaluation of caprylic acid against natural infections of *Sparicotyle chrysophrii* in cage-reared gilthead sea bream *Sparus aurata*. Aquaculture **408–409**, 15-19. (10.1016/j.aquaculture.2013.05.012)

[18] Rigos G, Mladineo I, Nikoloudaki C, Vrbatovic A, Kogiannou D. 2016 Application of compound mixture of caprylic acid, iron and mannan oligosaccharide against *Sparicotyle chrysophrii* (Monogenea: polyopisthocotylea) in gilthead sea bream, *Sparus aurata*. Folia Parasitol. **63**, 027. (10.14411/fp.2016.027) 27507773

[19] Firmino JP, Vallejos-Vidal E, Sarasquete C, Ortiz-Delgado JB, Balasch JC, Tort L, Estevez A, Reyes-López FE, Gisbert E. 2020 Unveiling the effect of dietary essential oils supplementation in *Sparus aurata* gills and its efficiency against the infestation by *Sparicotyle chrysophrii*. Sci. Rep. **10**, 17764. (10.1038/s41598-020-74625-5) 33082387 PMC7576129

[20] Repullés-Albelda A, Raga JA, Montero FE. 2011 Post-larval development of the microcotylid monogenean *Sparicotyle chrysophrii* (Van Beneden and Hesse, 1863): comparison with species of Microcotylidae and Heteraxinidae. Parasitol. Int. **60**, 512-520. (10.1016/j.parint.2011.09.008) 22015332

[21] Gurarie D, King CH, Wang X. 2010 A new approach to modelling schistosomiasis transmission based on stratified worm burden. Parasitology **137**, 1951-1965. (10.1017/S0031182010000867) 20624336 PMC3271124

[22] Gurarie D, King CH. 2015 Population biology of schistosoma mating, aggregation, and transmission breakpoints: more reliable model analysis for the end-game in communities at risk. PLoS ONE **9**, e115875. (10.1371/journal.pone.0115875) PMC4280120 25549362

[23] Gurarie D, King CH, Yoon N, Li E. 2016 Refined stratified-worm-burden models that incorporate specific biological features of human and snail hosts provide better estimates of schistosoma diagnosis, transmission, and control. Parasit. Vectors **9**, 428. (10.1186/s13071-016-1681-4) 27492409 PMC4973538

[24] Mari L, Gatto M, Ciddio M, Dia ED, Sokolow SH, De Leo GA, Casagrandi R. 2017 Big-data-driven modeling unveils country-wide drivers of endemic schistosomiasis. Sci. Rep. **7**, 489. (10.1038/s41598-017-00493-1) 28352101 PMC5428445

[25] Vrugt JA, Ter Braak CJF, Clark MP, Hyman JM, Robinson BA. 2008 Treatment of input uncertainty in hydrologic modeling: doing hydrology backward with Markov chain Monte Carlo simulation. Water Resour. Res. **44**, W00B09. (10.1029/2007WR006720)

[26] Vrugt JA, Ter Braak CJF, Diks CGH, Robinson BA, Hyman JM, Higdon D. 2009 Accelerating Markov Chain Monte Carlo simulation by differential evolution with self-adaptive randomized subspace sampling. Int. J. Nonlin. Sci. Numer. Simul. **10**, 273-290. (10.1515/IJNSNS.2009.10.3.273)

[27] Bush AO, Lafferty KD, Lotz JM, Shostak AW. 1997 Parasitology meets ecology on its own terms: Margolis et al. revisited. J. Parasitol. **83**, 575-583. (10.2307/3284227) 9267395

[28] Anderson RM, May RM. 1992 Infectious diseases of humans: dynamics and control. Oxford, UK: Oxford University Press.

[29] Ciddio M, Mari L, Gatto M, Rinaldo A, Casagrandi R. 2015 The temporal patterns of disease severity and prevalence in schistosomiasis. Chaos **25**, 036405. (10.1063/1.4908202) 25833443

[30] Lenhart S, Workman JT. 2007 Optimal control applied to biological models. New York, NY: CRC Press.

[31] Lemaitre JC, Pasetto D, Zanon M, Bertuzzo E, Mari L, Miccoli S, Casagrandi R, Gatto M, Rinaldo A. 2022 Optimal control of the spatial allocation of COVID-19 vaccines: Italy as a case study. PLoS Comput. Biol. **18**, e1010237. (10.1371/journal.pcbi.1010237) 35802755 PMC9299324

[32] Ferreira JG, Saurel C, Ferreira JM. 2012 Cultivation of gilthead bream in monoculture and integrated multi-trophic aquaculture. Analysis of production and environmental effects by means of the FARM model. Aquaculture **358–359**, 23-34. (10.1016/j.aquaculture.2012.06.015)

[33] Nobre AM, Valente LMP, Conceição L, Severino R, Lupatsch I. 2019 A bioenergetic and protein flux model to simulate fish growth in commercial farms: application to the gilthead seabream. Aquac. Eng. **84**, 12-22. (10.1016/j.aquaeng.2018.11.001)

[34] Ferreira JG, Taylor NGH, Cubillo A, Lencart-Silva J, Pastres R, Bergh Ø, Guilder J. 2021 An integrated model for aquaculture production, pathogen interaction, and environmental effects. Aquaculture **536**, 736438. (10.1016/j.aquaculture.2021.736438)

[35] Stella E, Bertuzzo E, Mari L. 2022 A stratified compartmental model for the transmission of *Sparicotyle chrysophrii*. Zenodo. (10.5281/zenodo.7236391) PMC10189595 37206963

